# Impact of an Antibiotic Stewardship Program on the Incidence of Resistant *Escherichia coli*: A Quasi-Experimental Study

**DOI:** 10.3390/antibiotics10020179

**Published:** 2021-02-10

**Authors:** Elad Ziv-On, Michael D Friger, Lisa Saidel-Odes, Abraham Borer, Orly Shimoni, Anna Nikonov, Lior Nesher

**Affiliations:** 1 Goldman Medical School, Faculty of Health Sciences, Ben-Gurion University of the Negev, Beer Sheva 84105, Israel; eladzivon88@gmail.com (E.Z.-O.); saidelod@bgu.ac.il (L.S.-O.); borer@bgu.ac.il (A.B.); 2 Department of Public Health, Faculty of Health Sciences, Ben-Gurion University of the Negev, Beer Sheva 84105, Israel; friger@bgu.ac.il; 3 Infectious Disease Institute, Soroka University Medical Center, Beer Sheva 84101, Israel; 4 Department of Pharmacy, Soroka University Medical Center, Beer Sheva 84101, Israel; orlyr@clalit.org.il (O.S.); annanik@clalit.org.il (A.N.)

**Keywords:** antibiotics selection pressure, antibiotics stewardship program, antibiotic use, *Escherichia coli* resistant strains, Gram-negative pathogens

## Abstract

Infections caused by multidrug-resistant Gram-negative bacteria increase hospitalizations and mortality rates; antibiotic pressure increases resistance rates. We evaluated the impact of the antibiotics stewardship program (ASP) on *Escherichia coli* resistance rates, evaluating all antibiotic use and patients with positive cultures hospitalized between 2011 and 2018. Data on antibiotics were collected quarterly as the defined daily dose (DDD)/100 days hospitalization. In 2014, an intervention was introduced, targeting the reduction of overall antibiotic use as well as specifically targeting quinolones and other broad-spectrum antibiotics. Using interrupted time series analysis (ITS), we compared the rates and trends of antibiotic use and resistant *E. coli*. We included 6001 patients, 3182 pre-ASP and 2819 post-ASP. We observed significant changes in absolute numbers as well as in trends for use of DDD/100 days of all antibiotics by 31% from 76 to 52, and by 52% from 10.4 to 4.9 for quinolones. ITS demonstrated that before the ASP intervention, there was a slope pattern for increased *E. coli* resistance to antibiotics. This slope was reversed following the intervention for quinolones −1.52, aminoglycosides −2.04, and amoxicillin clavulanate (amox/clav) −1.76; the effect of the intervention was observed as early as three months after the intervention and continued to decrease over time until the end of the study, at 48 months. We conclude that the ASP can positively impact the resistance rate of Gram-negative infections over time, regardless of the targeted combination of antibiotics, if the overall use is reduced.

## 1. Introduction

In recent years, bacterial resistance to antibiotics has increased globally. Multidrug-resistant (MDR) Gram-negative bacteria have become one of the greatest risks to public health [1,2], causing prolonged hospitalizations and higher death rates, especially when extended spectrum beta lactamase (ESBL) bacteria are involved. These pathogens almost double the risk of mortality compared to non-ESBL bacteria [3,4].

One of the major contributors to the increasing resistance rates is the unnecessary use of antibiotics, particularly broad-spectrum antibiotics, with reports of over 50% of antibiotics provided to patients without good cause [5,6]. Use of antibiotics causes selective pressure by killing susceptible bacteria, allowing antibiotic-resistant bacteria to survive and multiply. Broad-spectrum antibiotics are mostly used in hospitals, especially where patients with MDR infections are treated and are considered as having high antibiotic selection pressure. Unfortunately, despite repeated calls for action, the research and development of novel antimicrobial agents is not able to keep up with the development of bacterial resistance [7]. This has brought to the forefront the need to create Antibiotic Stewardship Programs (ASPs) for the improvement and utilization of existing antibiotics in hospitals and large healthcare systems [5,8,9,10,11,12].

The main goals of ASPs are to reduce the incidence of resistant infections [12,13] and to lower treatment costs while maintaining the quality of treatment [5,9]. To that end, ASPs focus mostly on reducing antibiotic selection pressure by restricting the overall use of antibiotics, treating for shorter periods, and choosing narrow-spectrum antibiotics as much as possible. In recent years, the Joint Commission International for hospital accreditation and regulatory bodies in various countries has required hospitals to set up an ASP as part of the accreditation process [14].

Although successful ASPs have reduced costs and improved resource utilization, there are limited data on their success in reducing the rate of resistant pathogens [15,16]. A meta-analysis published in 2017 reported a reduction in ESBL and MDR prevalence following implementation of an ASP, but did not find a significant reduction in the prevalence of quinolone resistance or aminoglycoside resistance in Gram-negative bacteria [13]. Penalva et al. noted a reduction in ESBL-producing *E. coli* in urine cultures following an educational community-based ASP intervention [17], and Peragine et al. reported a 9% reduction (incidence rate ratio of 0.91–0.99, *p* = 0.03) in hospital-acquired antibiotic-resistant organisms and a 13% (incidence rate ratio 0.87 0.73–1.04, *p* = 0.13) reduction in hospital-acquired MDR organisms [16].

Soroka University Medical Center (SUMC) is a 1100-bed tertiary/referral hospital, which serves as the only major medical provider for a population of approximately 900,000 people in the southern district of Israel. In recent years, we have observed a steady increase in Gram-negative-resistant infections in the southern district; in particular, we have seen a high prevalence of ESBL (~25% of urine pathogens) and MDR infections, which prompted an ASP intervention [18]. In this study, we aimed to evaluate the effect of an ASP intervention targeting the reduction of antibiotic selection pressure, specifically quinolones, on the resistance patterns of *E. coli* bacteria grown in urine and blood cultures.

## 2. Results

We collected medical records of 6001 cases: 3182 from the pre-ASP and 2819 from the post-ASP period. A total of 9387 positive *E. coli* bacterial cultures were identified: 5488 urine cultures and 505 blood cultures. Duplicates (*n* = 3394) were excluded. Baseline characteristics between the pre-ASP and post-ASP periods are compared in Table 1. Women provided 81% (4487/5488) and 56% (283/505) of urine and blood cultures, respectively (*p* < 0.001). The highest percentage of cultures was drawn in the emergency department (44.2% and 50.8% during the pre-ASP and post-ASP periods, respectively) and in the internal medicine department (30.7% and 27.5% during the pre-ASP and post-ASP periods, respectively). A significantly shorter length of hospital stay, from 4 to 3 days, was also noted between the pre-ASP and post-ASP periods (*p* < 0.001).

### 2.1. Change in Antibiotics Use during the Study

Following implementation of the ASP, overall use of antibiotics in the hospital significantly decreased by 31% from 76 defined daily doses (DDD)/100 bed days in the pre-ASP period to 51 DDD/100 bed days in the post-ASP period. Quinolone use decreased by 52% (from 10.4 to 4.9 DDD/bed 100 days), aminoglycoside use decreased by 32% (from 2.2 to 1.5 DDD/100 bed days), and amoxicillin clavulanate (amox/clav) use decreased by 58% (from 18.7 to 7.8 DDD/100 bed days). Antibiotic consumption by quarter is shown in Table S1.

**Figure 1:**
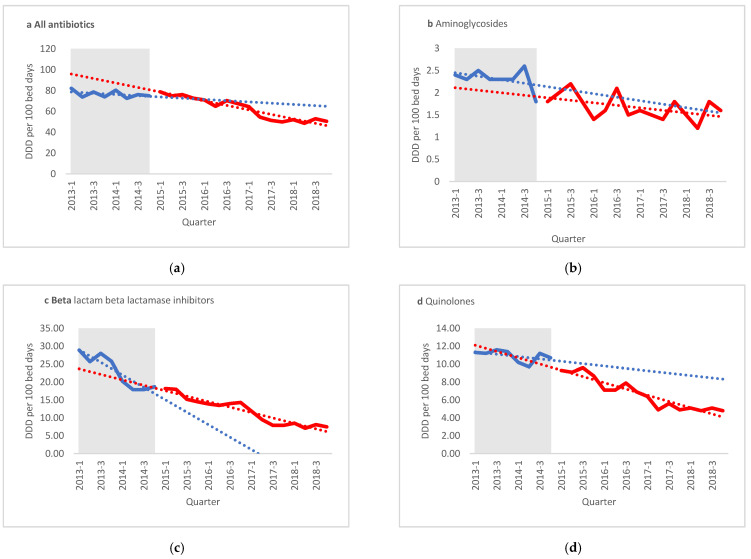

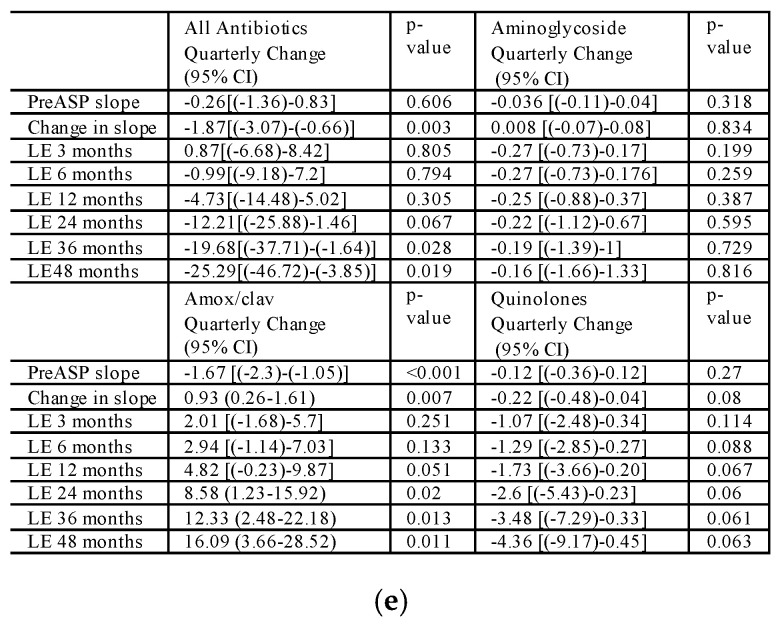
Interrupted time series analysis for quarterly defined daily doses (DDD)/100 bed days. (**a**) All antibiotics, (**b**) aminoglycosides, (**c**) beta lactam beta lactamase inhibitors, (**d**) quinolones. Shaded areas present pre-antibiotics stewardship intervention period. (**e**) LE—Level effect, PreASP—Pre-antibiotic stewardship intervention, Amox/clav—Amoxicillin clavulanate.

ITS analysis of antibiotic use showed that the ASP’s intervention significantly reduced the overall antibiotic use at SUMC from a slight negative slope of −0.26 to a strong negative slope of −1.87 (*p* = 0.003; Figure 1).

Aminoglycoside use decreased prior to the ASP intervention (a slope of −0.036) and maintained a negative slope throughout the study period, without any significant changes. A decreasing slope for use of amox/clav was noted before the intervention, with a negative slope of −1.67. This pattern continued, albeit mostly at a non-significantly slower rate.

### 2.2. Change in E. coli Resistance Rates to Antibiotics during the Study

**Figure 2:**
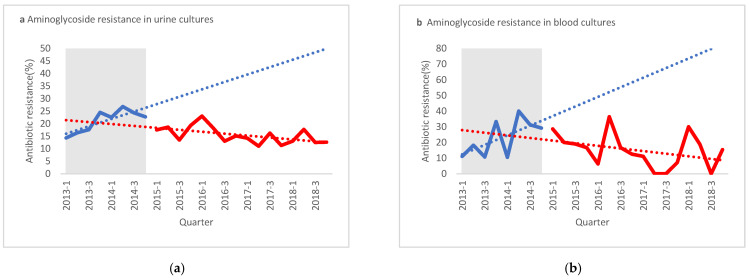

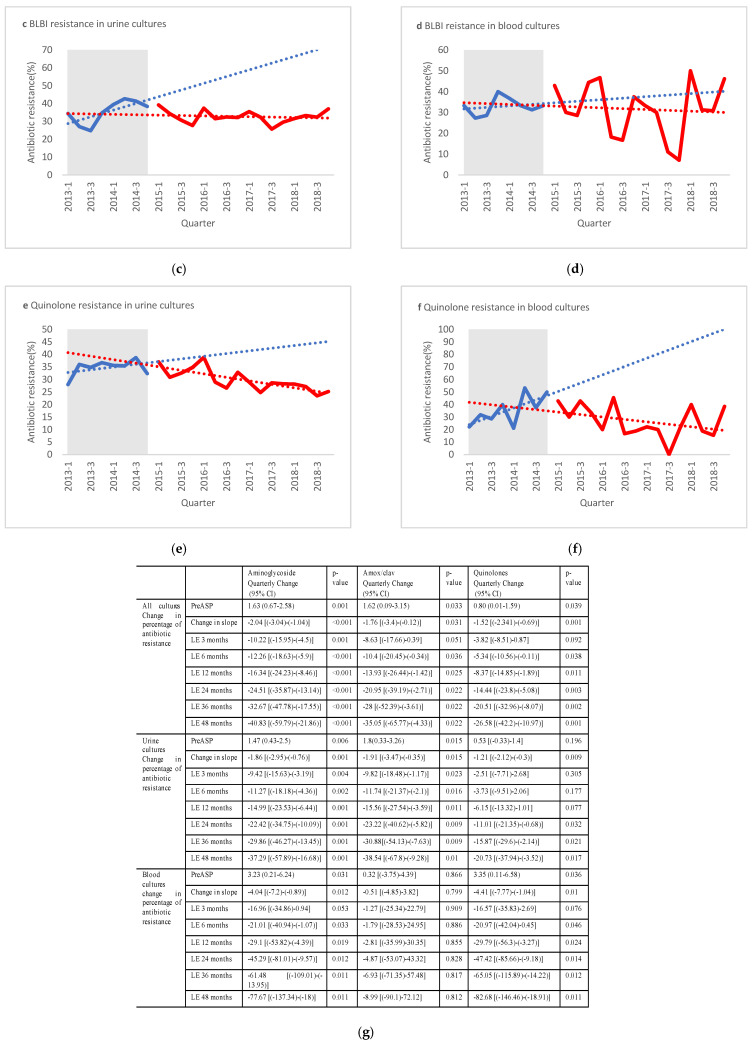
Interrupted time series analysis for antibiotic-resistant E. coli rates in blood and urine cultures. Aminoglycoside resistance in urine cultures (**a**) and blood cultures (**b**). Beta lactam/beta lactamase inhibitor (BLBLI) resistance in urine cultures (**c**) and blood cultures (**d**). Quinolone resistance in urine cultures (**e**) and blood cultures (**f**). Shaded areas present pre-antibiotics stewardship intervention period. (**g**) LE—Level effect, PreASP—Pre-antibiotic stewardship intervention, Amox/clav—Amoxicillin clavulanate.

As shown in Figure 2 and Table 2, cefuroxime and amox/clav resistance significantly increased in urine cultures between the pre-ASP and post-ASP periods (26.3% to 32.8%, *p* < 0.001; 29.6% to 32.6%, *p* = 0.015, respectively). In contrast, gentamicin and ciprofloxacin resistance significantly decreased in urine cultures between the pre-ASP and post-ASP periods (18.7% to 15.4%, *p* < 0.001; 33.6% to 30.1%, *p* < 0.005, respectively). A non-statistically significant decrease in resistance was also noted for ampicillin and trimethoprim/sulfamethoxazole in urine cultures.

In blood cultures, no statistically significant differences in antibiotic resistance were noted between the pre-ASP and post-ASP periods, probably due to the relatively small sample size. However, ITS analysis demonstrated a significant change in both slopes of resistance to aminoglycosides and quinolones from a positive increasing rate of resistance pre-ASP to a negative slope or decreasing rate of resistance post-ASP, a change in slope of −4.04 (*p* = 0.012) for aminoglycosides and −4.41 (*p* = 0.01) for quinolones (Figure 2).

## 3. Discussion

Our analysis showed that the ASP succeeded in both reducing the use of quinolones and reducing the overall use of antibiotics without increasing the use of nontargeted antibiotics. This reduction in antibiotic use lowered the rate of bacterial resistance to these drugs. Our results are similar to those reported by Boel [19] and Livermore [20], both of whom targeted cephalosporins and quinolones. However, Livermore reported increased use of beta-lactam/beta-lactamase inhibitors (a “squeezing the balloon effect”). In contrast, our study results show that targeting quinolone use without increasing the use of other antibiotics had the same effect on the reduction of resistance. This provides further evidence that ASP can positively impact the resistance rate of pathogens over time, regardless of the targeted combination of antibiotics, if overall use is reduced.

Our results support the concept that reduced exposure to antibiotics, particularly quinolones, has a positive long-term effect on resistance patterns. Quinolone use has been extremely high in recent years due to its excellent oral bioavailability, pharmacokinetic properties, and its activity spectrum against Gram-negative pathogens. However, in recent years, the use of quinolones has been targeted for reduction as a drug of interest for ASPs due to debilitating side effects as well as alarmingly increasing rates of resistance [21].

The overall use of amox/clav decreased by 50% during the study period. The observed decrease began early, even prior to the initiation of the ASP intervention. Although the reduction rate slowed over time, it continued to have a negative slope. Despite this decreased use rate, amox/clav resistance rates did not change. Since ESBL comprises several mechanisms that confer resistance to beta-lactams [22], reducing resistance may require longer periods of decreased antibiotic use until a change can be observed. It is also possible that reducing antibiotic use alone cannot impact resistance, requiring other multidisciplinary interventions, including infection control interventions and lowering of antibiotic selection pressure [23,24].

We chose *E. coli* resistance as the subject of intervention since it is one of the most common pathogens that causes clinically relevant infections. Furthermore, *E. coli* resistance is typically plasmid-mediated and, thus, can change rapidly. As such, it would be a marker for the early impact of antibiotic pressure changes resulting in a change in resistance patterns.

Although ASP intervention was analyzed by yearly quarters, it is a process, as seen in the long-lasting effects on the use of antibiotics. Our results are similar to those published by Penalva et al. [17], who showed that restriction of antibiotic dosage and dose reductions in the community setting were associated with a decreased prevalence of antibiotic-resistant bacteria. They also demonstrated a decreasing trend that began shortly (3–6 months) after the intervention was initiated and became stronger over time [17]. Our study confirms and strengthens other recent reports, such as that of Peragine et al. [16], who reported that ASPs have a crucial role in the fight to reduce MDR infections and have demonstrated long-term effects. Further resources need to be invested in ASP.

Our study has several limitations. First, this was a single-center study; however, SUMC’s remote location strengthens the proof that the reduction of antibiotic selection pressure has a positive effect on the rate of bacterial resistance. Second, data were collected only on antibiotic selection pressure at the hospital, while antibiotics are also prescribed in the community. Due to changing prescribing patterns and the fact that quinolones were designated as a second-line drug by the US Food and Drug Administration (FDA), a black box warning was added in 2016 [25], which may have influenced the use of antibiotics in the community and, therefore, the antibiotic resistance pattern. However, this change was observed in the period prior to the publication of the black box warning, so it probably had an additive effect. Last, most of our cohort comprised women, due to the higher prevalence of urinary infections in women compared to men. Although this gender difference disappeared when blood cultures were analyzed, the ASP may have had a greater impact on women than on men.

## 4. Materials and Methods

### 4.1. Intervention

In the fourth quarter of 2014, we formed an ASP team comprising an infectious disease physician and two clinical pharmacists, and a hospital-wide intervention was implemented in 2015. The intervention included educational lectures on the ASP to medical staff, including nurses, residents, and attending physicians in each ward. In addition, a computerized authorization form for restricted antibiotics, which had to be renewed every three days (automatic stop order), was introduced. Restricted antibiotics included all antibiotics except for penicillin, metronidazole, 1st and 2nd generation cephalosporins, and 3rd generation ceftriaxone. All restricted antibiotics prescribed were authorized by an infectious disease specialist and then reviewed by a dedicated clinical pharmacist for adjustment to renal function, interactions with other medications, and appropriate dosing for the intended use. Each department head in the hospital received daily feedback by electronic notification of all patients under their care that had been treated with antibiotics for more than 5 days. All department heads were required to include, in their yearly reports to management, the rates of antimicrobial consumption using defined daily doses (DDD)/100 bed days compared to that of previous years. The program did not change any existing treatment protocols or practices of restricting antibiotics; however, it aimed at overall reduction of antibiotics use, particularly targeting a reduction in the use of quinolones and amoxicillin/clavulanate (amox/clav).

### 4.2. Study Design and Patients

The study was approved by SUMC’s ethics committee, with ID Sor-19-008. The requirement for a patient’s informed consent was waived. The study was designed and reported utilizing STROBE-AMS methodology [26]. Clinical records of all adult patients that were admitted to SUMC between January 2011 and December 2018 and included data on *E. coli* cultures in blood or urine were included in the analysis.

### 4.3. Data Sources and Collection

Patient data were collected retrospectively from SUMC’s electronic medical records.

All urine and blood cultures were performed by SUMC’s Microbiology Laboratory Service according to standard techniques. Antimicrobial susceptibility tests were performed using the Kirby–Bauer disk diffusion method [27] or the VITEK^®^ 2 microbial identification system and antibiotic susceptibility testing (bioMerieux SA, Marcy l’Etoile, France). Interpretation of test results was performed according to the guidelines proposed by the Clinical Laboratory Standards Institute (CLSI), which are updated yearly [28].

For the purpose of analysis, culture results that included a sensitivity panel and were defined as intermediate or resistant according to CLSI standards were classified as resistant. Since the SUMC lab only began to electronically mark cultures as ESBL-positive in 2016, we were unable to collect this information for the whole study period. To prevent duplication, the first positive culture was chosen for each admission.

Data on antibiotic use were collected from SUMC’s bioinformatics systems, which collects information from patients’ electronic medical records as well as from logistic and supply sources. Data on antibiotic consumption are presented per annual quarter as DDD per 100 days of admission.

### 4.4. Outcome Measures

The percentage of resistant strains in urine and blood cultures was compared for each antibiotic between the pre-ASP—from the beginning of Q1 2013 (January 2013) to the end of Q4 2014 (December 2014)—and post-ASP—from the beginning of Q1 2015 (January 2015) to the end of Q4 2018 (December 2018)—periods. All quarterly antibiotic use per DDD/100 bed days beginning in the first quarter of 2013 (data were not available prior this date) and up to the fourth quarter of 2018 was analyzed.

### 4.5. Statistical Analysis

Categorical variables were summarized as frequencies and percentages, and continuous variables were summarized as mean and standard deviation (SD) or median with interquartile range as appropriate. Chi-squared test or Fisher’s exact test were employed for comparing categorical nominal variables. Continuous values were compared using the unpaired Student *t* test for normally distributed data, or the Mann–Whitney U test for data without a normal distribution. The prevalence of resistant *E. coli* was examined for each antibiotic, by the percentage of total positive *E. coli* found in the same period. To examine the effect of the intervention on DDD and antibiotic resistance over time, interrupted time series analysis (ITS) was performed. ITS analysis is a quasi-experimental design, which uses Autoregressive Integrated Moving Average models. This statistical method investigates time trends before and after an intervention. The ITS analysis was based on the quarterly DDD value for each antibiotic that was consumed in SUMC, in addition to the quarterly percentage of antibiotic resistance, calculated for each antibiotic. We examined the trend of the slope before and after the intervention; furthermore, we examined the level effect—specifically, the change in level for every period comprised with the preintervention predicted value. Statistical calculations were performed using IBM SPSS Statistics for Windows, Version 25.0 (IBM Corp., Armonk, NY, USA). A *p*-value less than 0.05 was considered statistically significant.

## 5. Conclusions

In conclusion, an ASP that targets antibiotic selection pressure is an effective way to impact the resistance patterns of Gram-negative infections, specifically *E. coli*. This is an important affirmation that investing in ASP will have a clinical impact in decreasing antibiotic-resistant bacteria.

## Figures and Tables

**Table 1:** Description of the patients included in study. Each patient was included only once per admission.

Variable	All Cultures			Urine Cultures			Blood Cultures		
	Pre-ASP *n* = 3182	Post-ASP *n* = 2819	*p*-value	Pre-ASP *n* = 2897	Post-ASP *n* = 2591	*p*-value	Pre-ASP *n* = 284	Post-ASP *n* = 221	*p*-value
Gender, *n* (%)									
Male	689 (21.7)	533 (19)	0.009	562 (19.4)	438 (16.9)	0.017	127 (44.7)	95 (43)	0.697
Female	2491 (78.3)	2279 (81)		2334 (80.6)	2153 (83.1)		157 (55.3)	126 (57)	
Age, years, median (IQR 25, 75)	70 (40, 83)	63 (30, 81)	<0.001	69 (39, 82)	61 (29, 81)	<0.001	73.5 (56, 84)	71 (57, 85)	0.82
Internal medicine	976 (30.7)	773 (27.5)	<0.001	910 (31.4)	727 (28.1)	<0.001	66 (23.2)	46 (20.8)	0.1
Surgery	319 (10)	225 (8)		296 (10.2)	208 (8)		23 (8.1)	17 (7.7)	
Gynecology	254 (8)	214 (7.6)		243 (8.4)	212 (8.2)		11 (3.9)	2 (0.9)	
Emergency department	1407 (44.2)	1429 (50.8)		1249 (43.1)	1285 (49.6)		158 (55.6)	144 (65.2)	
Intensive care unit	143 (4.5)	99 (3.5)		121 (4.2)	90 (3.5)		22 (7.7)	9 (4.1)	
Other	82 (2.6)	72 (2.6)		78 (2.7)	69 (2.7)		4 (1.4)	3 (1.4)	
Length of hospitalization, Days, median (IQR 25, 85)	4 (2, 8)	3 (1, 7)	<0.001	4 (1, 8)	3 (0, 6)	<0.001	7 (3, 14)	6 (3, 11)	0.265

**Table 2:** Antibiotic resistance of E. coli in urine and blood cultures before and after ASP intervention.

Antibiotic	All Cultures Resistant/Tested (%)			Urine Cultures Resistant/Tested (%)			Blood Cultures Resistant/Tested (%)		
	Pre-ASP *n* = 3182	Post-ASP *n* = 2819	*p* value	Pre-ASP *n* = 2897	Post-ASP *n* = 2591	*p* value	Pre-ASP *n* = 284	Post-ASP *n* = 221	*p* value
Ampicillin	2256/3174 (71.1)	1923/2796 (68.8)	0.053	2059/2896 (71.1)	1775/2577 (68.9)	0.074	197/277 (71.1)	143/212 (67.4)	0.383
Cefuroxime	842/3174 (26.5)	943/2788 (33.8)	<0.001	762/2896 (26.3)	845/2570 (32.8)	<0.001	80/277 (28.8)	66/211 (31.2)	0.566
Gentamycin	603/3175 (19)	430/2796 (15.4)	<0.001	543/2897 (18.7)	398/2577 (15.4)	0.001	60/277 (21.6)	32/212 (15.1)	0.066
Amox/clav	943/3175 (29.7)	910/2788 (32.6)	0.014	857/2897 (29.6)	839/2570 (32.6)	0.015	86/277 (31)	68/211 (32.2)	0.781
Ciprofloxacin	1074/3175 (33.8)	833/2787 (29.9)	0.001	975/2897 (33.6)	773/2569 (30.1)	0.005	99/277 (35.7)	58/211 (27.5)	0.053
Tmp/Smx	1199/3175 (37.8)	993/2787 (35.6)	0.075	1093/2897 (37.7)	911/2576 (35.4)	0.07	106/277 (38.3)	81/211 (38.4)	0.978

ASP—Antibiotic Stewardship Program; amox/clav—amoxicillin clavulanate, Tmp/Smx—trimethoprim sulfamethoxazole.

## Data Availability

The datasets generated and/or analyzed during the current study are being used for further research and are available from the corresponding author on reasonable request.

## Supplementary Materials

The following are available online at https://www.mdpi.com/2079-6382/10/2/179/s1, Table S1: Quarterly use of antibiotics in the hospital by DDD/100 days of admission.
